# Vitamin D Levels and SARS-CoV-2 Infection among Medically Underserved Populations in the Minority and Rural Coronavirus Insights Study

**DOI:** 10.3390/v16040639

**Published:** 2024-04-19

**Authors:** Makella S. Coudray, Shantoy Hansel, Salvatore Alesci, William A. Meyer, Robert H. Christenson, Latrice G. Landry, Christina Edwards, Gary Puckrein, Derrick J. Forney, Ola Akinboboye

**Affiliations:** 1 National Minority Quality Forum, 1201 15th St NW #340, Washington, DC 20005, USAsalesci@nmqf.org (S.A.); gpuckrein@nmqf.org (G.P.); dforney@nmqf.org (D.J.F.); 2 Department of Population Health Sciences, College of Medicine, University of Central Florida, 6900 Lake Nona Blvd, Orlando, FL 32827, USA; 3 Quest Diagnostics, 500 Plaza Dr, Secaucus, NJ 07094, USA; william.a.meyer@questdiagnostics.com; 4 Department of Pathology, University of Maryland School of Medicine, 655 W. Baltimore St., Baltimore, MD 21201, USA; rchristenson@som.umaryland.edu; 5 Perelman School of Medicine, University of Pennsylvania, 3400 Civic Center Blvd, Building 421, Philadelphia, PA 19104, USA; 6 Queens Heart Institute, 23436 Merrick Blvd, New York, NY 11422, USA; akinboboye@gmail.com

**Keywords:** COVID-19, SARS-CoV-2, vitamin D, health disparities

## Abstract

Background: Extant literature presents contradictory findings on the role of vitamin D on SARS-CoV-2 infection. Our study included an examination of the relationship between vitamin D levels and SARS-CoV-2 infection among the Minority and Rural Coronavirus Insights Study (MRCIS) cohort, a diverse population of medically underserved persons presenting at five Federally qualified health centers in the United States. Methods: We conducted a descriptive analysis to explore the relationship between vitamin D levels and SARS-CoV-2 infection among medically underserved participants. A combined molecular and serologic assessment was used to determine the prevalence of SARS-CoV-2 infection. Vitamin D was examined as both a categorical (vitamin D status: deficient, insufficient, optimal) and continuous (vitamin D level) variable. Chi-squared testing, polynomial regression models, and logistic regression models were used to assess the relationship between vitamin D and SARS-CoV-2 infection. Results: The overall SARS-CoV-2 infection rate among participants was 25.9%. Most participants were either vitamin D deficient (46.5%) or insufficient (29.7%), and 23.8% had an optimal level. Vitamin D status was significantly associated with key SARS-CoV-2 infection risk factors. As mean vitamin D levels increased, the proportion of participants with SARS-CoV-2 infection decreased. For every 10 ng/mL increase in vitamin D levels the odds of SARS-CoV-2 infection decreased by 12% when adjusting for race/ethnicity and age (main effect model). Participants who identified as Hispanic/Latino or Black non-Hispanic had approximately two times increased odds of SARS-CoV-2 infection when adjusting for age and vitamin D levels compared to white non-Hispanics. However, when additional factors were added to the main effect model, the relationship between vitamin D levels and SARS-CoV-2 infection did not remain significant. Conclusion: Vitamin D levels were associated with an increased risk of SARS-CoV-2 infection. Hispanic/Latino and Black, non-Hispanic compared to White, non-Hispanic participants were at increased odds for infection, after adjusting for race/ethnicity and age.

## 1. Introduction

The COVID-19 pandemic has unmasked profound racial and ethnic health disparities, a phenomenon well documented in extant literature. Racial and ethnic minority groups have been adversely and disproportionately impacted by the pandemic, experiencing higher rates of infection, severe disease, and mortality [1]. These disparities extend beyond genetic predispositions and are intricately linked to the social determinants of health, including differential access to healthcare, socioeconomic inequities, and disparate living conditions [2]. In the realm of mitigating factors, vitamin D has emerged as a variable of interest in the epidemiological landscape of COVID-19. Known for its immunomodulatory and immunostimulatory properties, vitamin D plays a pivotal role in immune response regulation [3].

Epidemiological studies have consistently highlighted vitamin D deficiency as a prevalent issue among racial and ethnic minorities, a disparity caused by factors such as skin pigmentation and dietary variations [4,5,6]. Vitamin D deficiency has been associated with conditions such as diabetes, rheumatoid arthritis, and acute respiratory tract infections [7]. The association between vitamin D status and COVID-19 outcomes has garnered significant attention in recent research. Some studies indicate a potential clinically significant association between vitamin D deficiency and increased severity of COVID-19 symptoms [8]. Conversely, adequate vitamin D levels or supplementation have been associated with less severe disease courses in some cohorts [9]. This evidence suggests a possible role of vitamin D in modulating the impact of COVID-19, particularly among populations already at elevated risk due to systemic health disparities.

Vitamin D supplementation, an easily accessible (over the counter), predominantly safe, and affordable intervention, could be an important tool to assist in the prevention of SARS-CoV-2 infection among those facing the highest burden, (i.e., racial/ethnic minority groups). Extant literature presents complex and sometimes contradictory findings on the relationship between vitamin D level and SARS-CoV-2 infection [5]. Some research indicates that vitamin D deficiency may increase the risk of serious SARS-CoV-2 infection, while others indicate that vitamin D was not associated with a higher risk of infection [10,11]. These discrepancies may be attributed to varying study designs (cross-sectional vs. longitudinal), different classifications of vitamin D deficiency (multiple standards exist), and difference in sample population (minority populations vs. predominantly White populations). Additionally, there is a lack of an examination of the role of pre-existing comorbidities and geographic location on the relationship between vitamin D and SARS-CoV-2 infection. This inconsistency underscores the need for a nuanced understanding of the multifaceted relationship between vitamin D levels, SARS-CoV-2 infection, and the social determinants of health, especially in racially and ethnically diverse populations. Our study aims to fill this gap by focusing on this historically marginalized population.

People in medically underserved communities have a higher proportion of racial/ethnic minorities—the groups that are disproportionally affected by COVID-19 [12], vitamin D deficiency [13,14], and comorbidities [15]—which may modify the relationships between vitamin D and susceptibility to SARS-CoV-2 infection. Here we present the results from the Minority and Rural Coronavirus Insights Study (MRCIS) conducted among medically underserved communities across five US states [1]. MRCIS is the largest multi-site, community-based, epidemiologic investigation of the social and structural drivers of health, clinical, environmental, and genetic factors associated with the COVID-19 pandemic in minority and rural communities in the US. Here, we describe the relationships between circulating vitamin D levels and the evidence of SARS-CoV-2 infection in this largely vaccine-naïve study population. This investigation is crucial for informing public health strategies and interventions tailored to the needs of diverse populations.

## 2. Methods

We conducted a descriptive analysis to examine the relationship between circulating serum vitamin D levels and SARS-CoV-2 infection among the MRCIS cohort. MRCIS aimed to examine various physiological and social demographic factors associated with the molecular and serological assessment of SARS-CoV-2 infection. A detailed description of the study methods and cohort has been published elsewhere [1].

### 2.1. Study Enrollment

Briefly, MRCIS was launched as a five-year cohort study by the National Minority Quality Forum (NMQF) to examine risk factors associated with the disproportionate impact of COVID-19 on minority and rural communities. The study was initiated in November 2020, and follow-up procedures will be completed in April 2026. All participants were recruited between November 2020 and April 2021. Through NMQF’s existing partnerships, Federally Qualified Health Centers (FQHCs), funded through the Health Resources & Services Administration (HRSA), were invited to participate in MRCIS as community-based healthcare providers. FQHCs primarily provide services for underserved communities that have historically been underrepresented in clinical research investigations. Thus, the inclusion of FQHCs was instrumental in ensuring the participation of medically underserved communities. Participants were recruited from five FQHCs: Osceola Community Health Services (Kissimmee, FL, USA), Teche Action Clinic (Franklin, LA, USA), John Wesley County Hospital (Los Angeles, CA, USA), Aunt Martha, (Olympia Fields, IL, USA), and Primary One (Columbus, OH, USA) [1]. These specific FQHCs were selected given their access to minority populations and/or their rural site designation by HRSA.

Convenience sampling techniques were implemented to recruit adult (18 years and older) volunteers. Multiple recruitment strategies were implemented: in-person recruitment (at study health centers, recreational centers, low-income housing complexes, fire departments, and homeless shelters) and by advertising through marketing flyers distributed to the community. Eligible volunteers completed an informed consent form and a baseline social demographic survey, and underwent sample collections. The study was approved by the WIRB-Copernicus Group Institutional Review Board (WCG IRB) under protocol number [#1292174].

### 2.2. Data Collection

The baseline survey assessed demographic information (e.g., race, ethnicity, age, sex), social drivers of health (e.g., access to utilities, access to transportation, housing instability), COVID-19 mitigation behaviors and practices (e.g., social distancing, mask-wearing), and medical history (i.e., presence of symptoms and comorbidities). Study coordinators involved in data collection received rigorous training on data collection techniques to improve data quality and completeness.

#### Laboratory Measurements

At enrollment, clinical research staff obtained nasal swabs from participants for a SARS-CoV-2 RNA nucleic acid amplification test (NAAT) and a peripheral venous blood specimen, collected in EDTA-anticoagulated and serum separator evacuated tubes. Laboratory testing was performed through the regional Quest Diagnostics laboratory facility that serviced each FQHC clinical research site. All biological specimens were collected on the same day.

Main Outcome: The SARS-CoV-2 IgG antibody testing was performed using one of the following US Food and Drug Administration Emergency Use Authorization testing platforms: (i) Abbott Architect SARS-CoV-2 IgG (Abbot Park, IL, USA); or (ii) Ortho-Clinical Diagnostics VITROS Anti-SARS-CoV-2 IgG test (San Diego, CA, USA). Anterior nasal swab sample SARS-CoV-2 RNA NAAT testing used one of the following US Food and Drug Administration Emergency Use Authorization testing platforms: (i) a Quest Diagnostics laboratory developed test modeled on the US Centers for Disease Control and Prevention (CDC) methodology, (ii) Cobas^®^ (Roche Molecular Systems, Inc., Pleasanton, CA, USA), (iii) Panther Fusion, (Hologic, Inc., Marlborough, MA, USA), or (iv) Aptima (Hologic, Inc., Marlborough, MA, USA). Prevalence of SARS-CoV-2 infection was determined as a positive result for either or both molecular or serologic assessments. If at the time of specimen collection a participant was eligible for the COVID-19 vaccine based on age requirements by state, then only the (i) SARS-CoV-2 RNA NAAT and (ii) Abbott Architect SARS-CoV-2 nucleocapsid-protein IgG test results were used to assess SARS-CoV-2 infection status.

Main Exposure: The circulating level of vitamin D was assessed using the FDA-cleared total 25 hydroxyvitamin D 25(OH)D Atellica^®^ Solution immunoassay method from Siemens Healthineers (Malvern, PA, USA).

### 2.3. Statistical Analysis

Descriptive analysis was conducted to examine all variables. Circulating vitamin D, self-reported age, and number of comorbidities were the only continuous variables included in the analysis and were summarized using mean, median, standard deviation, range, interquartile range, and skewness. The linearity of the association between SARS-CoV-2 and vitamin D was examined by plotting the prevalence of infection in each decile. Vitamin D levels were further categorized into three groups based on existing guidelines [16]: deficient—<20 ng/mL, insufficient—≥20 and <30 ng/mL, and optimal—≥30 ng/mL. Participant age was further categorized into three groups: 18–39, 40–59, and ≥60 years. Number of self-reported comorbidities was further categorized into four groups: 0, 1–2, 3–4 and ≥5. Frequencies and percentages were calculated for all categorical variables. Race and ethnicity information was categorized into four mutually exclusive groups: Hispanic/Latino, non-Hispanic Black/African American, non-Hispanic White, and non-Hispanic Other. A “region” variable was created to recategorize the five study sites: Southeast (Osceola Community Health Services, and Teche Action Clinic), Midwest (Aunt Martha, and Primary One), and West (John Wesley County Hospital).

The study population characteristics were compared by vitamin D status (deficient: <20 ng/mL, insufficient: 20–30 ng/mL, optimal: >30 ng/mL) and SARS-CoV-2 infection status (positive, negative) using Chi-squared testing. Logistic regression models were used to assess the adjusted odds ratios (ORs) and their 95% confidence intervals (CIs). Due to the low number of respondents in certain categories, the sample used for logistic regression was restricted to exclude the following categories: ethnicity—“non-Hispanic Other” and sex—“Other”. Additionally, due to the violation of the assumption of linearity (relationship between vitamin D levels and SARS-CoV-2 infection), participants in the tenth decile of vitamin D levels were removed for the logistic regression analysis. The main effect model included age, race/ethnicity, and vitamin D level (scaled to the 10 ng/mL interval). The referent group race/ethnicity was White non-Hispanic. The main effect model was then used to assess the strength of the association of the remaining variables by individually adding each variable to the main effect model. Data were analyzed using SAS version 9.4 (SAS Institute, Inc., Cary, NC, USA). Data visualization was carried out using R and R Studio version 4.3.1 (Boston, MA, USA).

## 3. Results

The final analytical sample included 3187 participants for whom vitamin D level test results and SARS-CoV-2 test results ((i) SARS-CoV-2 RNA NAAT, (ii) Abbott Architect SARS-CoV-2 IgG or (iii) Ortho-Clinical Diagnostics VITROS Anti-SARS-CoV-2 IgG) were available (Supplementary Materials, Figure S1). The study population included male (39.5%) and female (60.5%) adults (age range: 18 to 96 years, mean age = 50.3 years, standard deviation = 15.8 years), who identified as Black non-Hispanic (25.2%), Hispanic or Latino (48.9%), Other (2.6%), or White non-Hispanic (20.1%) (Table 1). Of these, 825 (25.9%) tested positive for SARS-CoV-2 infection (Table 1). Most participants were classified as either vitamin D deficient (46.5%) or insufficient (29.7%), and the remaining 23.8% of participants had optimal vitamin D levels (Table 1). Bivariate analysis demonstrated a statistically significant (*p* ≤ 0.05) relationship between vitamin D categorical status and SARS-CoV-2 infection (Table 1). Vitamin D status was significantly associated with key SARS-CoV-2 infection risk factors such as age (*p* < 0.01), race and ethnicity (*p* < 0.01), pre-existing co-morbidities (number of comorbidities (*p* < 0.01) or pre-existing cardiometabolic co-morbidities (*p* < 0.01)), and geographic location (residing in a rural setting (*p* < 0.01) or residing in the West, Midwest, or Southeast (*p* < 0.01)) (Table 2). Medical history such as diabetes (*p* < 0.01), history of heart attack (*p* = 0.02), high blood pressure (*p* < 0.01), and cardiac conditions (*p* < 0.01) were also associated with vitamin D status (Table 3).

**Figure 1:**
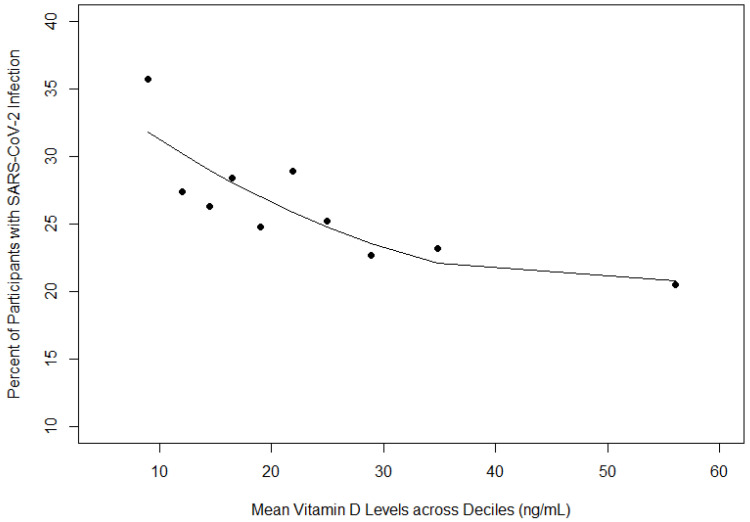
Prevalence of SARS-CoV-2 infection by decile of vitamin D distribution. The trend line represents a second-degree polynomial. Adjusted R^2^ = 0.62.

Vitamin D was also examined as a continuous variable. As mean vitamin D levels increased across deciles (one through nine), the proportion of participants with SARS-CoV-2 infection decreased (Figure 1). Racial and ethnic differences were also apparent (Table 4). White non-Hispanic participants reported the highest mean levels of vitamin D (26.3 ± 23.0) and had the lowest proportion of SARS-CoV-2 infection (15.6%) (Table 4). Black non-Hispanic participants reported the second highest mean levels of vitamin D (25.5 ± 14.9) and had the second highest proportion of SARS-CoV-2 infection (26.7%) (Table 4). Hispanic participants reported the lowest mean levels of vitamin D (21.9 ± 12.1) and had the highest proportion of SARS-CoV-2 infection (29.5%) (Table 4). Logistic regression results indicated that for every 10 unit increase in vitamin D levels, the odds of SARS-CoV-2 infection decreased by 12% when adjusting for race/ethnicity and age (Table 5). Additionally, participants who identified as Hispanic/Latino or Black non-Hispanic had approximately two fold increased odds of SARS-CoV-2 infection when adjusting for age and vitamin D levels (odds ratio (OR): 2.01, confidence interval (CI): 1.56–2.59 (Hispanic/Latino vs. White non-Hispanic) and OR: 1.91, CI: 1.44–2.53 (Black non-Hispanic vs. White non-Hispanic)) (Table 5). Age had minimal effect on the relationship between vitamin D levels and SARS-CoV-2 infection (OR: 0.99, CI: 0.98–0.99) (Table 5). This main effect model was also analyzed using categorical vitamin D status and the relationship between vitamin D status and SARS-CoV-2 infection was not significant (Supplementary Materials, Table S1).

The main effect model (age, race/ethnicity, and vitamin D level (scaled to the 10 ng/mL interval); Table 5) was used to independently assess the relationship between region and the presence of pre-existing comorbidities and SARS-CoV-2 infection. Vitamin D levels varied by region, with the highest levels in the Southeast and the lowest in the West (Table 6). When adjusting for age, race/ethnicity, and vitamin D levels, the region was significantly associated with SARS-CoV-2 infection (OR: 4.04, CI: 3.08–5.28 (Midwest vs. Southeast); OR: 4.51, CI: 3.65–5.57 (West vs. Southeast)); however, vitamin D did not remain significant in this model (Table 6). Residing in a rural setting was significantly associated with SARS-CoV-2 infection (OR: 0.45, CI: 0.35–0.58), however, vitamin D did not remain significant in this model (Table 6). Conversely, when adjusting for age, race/ethnicity, and vitamin D levels neither cardiometabolic outcomes (OR: 1.03, CI: 0.84–1.26) nor the number of comorbidities were significantly associated with SARS-CoV-2 infection (OR: 1.04, CI: 0.85–1.26 (1–2 comorbidities vs. 0 comorbidities); OR: 0.74, CI: 0.54–1.03 (3–4 comorbidities vs. 0 comorbidities); OR: 0.86, CI: 0.45–1.63 (≥5 comorbidities vs. 0 comorbidities)) (Table 6). Interaction terms were not significant (region × vitamin D, rural setting × vitamin D).

## 4. Discussion

This study demonstrated that among a large population of medically underserved persons, for every 10 unit increase in vitamin D levels the odds of SARS-CoV-2 infection decreased by 12%. Our findings are consistent with extant literature that has demonstrated that vitamin D deficiency increases the risk of SARS-CoV-2 infection [17]. Kaufman et al. conducted a large retrospective analysis that included more than 190,000 patients from all 50 states [4]. They concluded that SARS-CoV-2 infection was significantly associated with vitamin D levels, i.e., as vitamin D levels decreased SARS-CoV-2 positivity rates increased [4]. This relationship was similar across all racial/ethnic groups in the sample. Further examination is suggested of the effectiveness of vitamin D supplementation as a prevention method for SARS-CoV-2 infection and in the management of disease progression. Both the affordability and the availability of vitamin D add to the allure of such a strategy.

We also concluded that racial/ethnic minority groups had lower levels of vitamin D and two times increased odds of SARS-CoV-2 infection compared to White non-Hispanics. These differences may be a result of socioeconomic, nutritional, and/or genetic factors that might influence these outcomes. African Americans individuals experience increased burden of many COVID-19 risk factors such as lower socioeconomic status and higher rates of comorbidities such as diabetes and cardiovascular disease [18]. Additionally, African American people experience lower serum vitamin D levels [19]. Our findings are consistent with those of Rodriguez and colleagues who concluded that Hispanics experience increased odds of SARS-CoV-2 infection [20].

Of note, though the association between vitamin D levels and SARS-CoV-2 infection was significant in the main effect model (SARS-CoV-2 infection = age + race/ethnicity + vitamin D level), this association did not remain significant when additional variables (region, residing in a rural setting, cardiometabolic outcomes, number of comorbidities) were added to the main effect model. Interaction terms (region × vitamin D, rural setting × vitamin D) were not statistically significant. The non-significance of vitamin D levels in the minimally adjusted models could potentially be attributed to unmeasured factors such as the degree of sun exposure that directly impact vitamin D levels and vary by region. Thus, the importance of vitamin D’s role in SARS-CoV-2 infection must be interpreted carefully within the context of varying geographic settings. Additionally, there was minimal effect of age on the relationship between vitamin D levels and SARS-CoV-2 infection. Given the findings in extant literature that demonstrates age-related immune function changes and increases in the risk of SARS-CoV-2 infection among older adults [21], this finding warrants further examination in future studies.

The findings presented here should be interpreted thoughtfully considering the study’s limitations. The cross-sectional study design did not allow for examination of the direction of the relationship between vitamin D levels and SARS-CoV-2 infection. The study survey did not capture data about vitamin D supplementation; thus, the effect of this variable could not be assessed. Furthermore, participant recruitment for MRCIS occurred over a six-month period across five states. Differences in daylight exposure were not accounted for. The use of convenience sampling, though a suitable technique at the time of recruitment, may have resulted in sampling bias and limited the external validity of the study’s findings. These findings may not be applicable to persons who seek care at facilities other than FQHCs and do not face barriers to accessing care. Due to the limitations in the sample size, participants in the “Other” racial/ethnic group were not included in the final regression analyses to maintain statistical power and achieve model convergence. This did now allow for detailed examination of significantly understudied populations which includes American Indians/Alaskan Natives and Native Hawaiians/Other Pacific Islanders. Despite this limitation, the MRCIS cohort includes a predominantly racially and ethnically diverse sample and is one of the only longitudinal studies that examines COVID-19 in this medically underserved population that includes serologic assessment of markers of disease. The longitudinal nature of MRCIS also allows for future research to examine potential changes in vitamin D levels and the relationship with COVID-19, an area where there is a significant paucity of research.

Future research examining the relationship between vitamin D levels and COVID-19 should consider longitudinal study designs to assess the potential causal influence of vitamin D. Additionally, intervention trials are needed to further elucidate the effects of vitamin D supplementation as a preventive measure for COVID-19 and its effects as a component of COVID-19 treatment plan [22].

## 5. Conclusions

When demographic factors (age and race/ethnicity) were considered, vitamin D levels significantly reduced the odds of SARS-CoV-2 infection. However, this relationship was not significant when factors such as geographic location (region and rural setting) were considered. Data included in the study did not allow for an examination of varying levels of sun exposure by geographic location which may have impacted the relationship between vitamin D levels and SARS-CoV-2 infection, and this should be examined in future research. 

## Figures and Tables

**Table 1:** Relationships between cohort characteristics and the combined molecular and serologic assessment of SARS-CoV-2 status (*n* = 3187).

Variables	Total	SARS-CoV-2 Status		*p*-Value
		Positive (*n* = 825)	Negative (*n* = 2362)	
	N (%)	N (%)	N (%)	
**Age**				**<0.01**
18–39	871 (27.3)	243 (29.5)	628 (26.6)	
40–59	1333 (41.8)	393 (47.6)	940 (39.8)	
60+	983 (30.8)	189 (22.9)	794 (33.6)	
**Sex**				**0.03**
Female	1915 (60.1)	483 (58.6)	1432 (60.6)	
Male	1260 (39.5)	335 (40.6)	925 (39.2)	
Other	3 (0.1)	1 (0.1)	2 (0.1)	
**Race and Ethnicity**				**<0.01**
Hispanic or Latino	1558 (48.9)	460 (55.8)	1098 (46.5)	
Black, non-Hispanic	802 (25.2)	214 (25.9)	588 (24.9)	
White, non-Hispanic	640 (20.1)	100 (12.1)	540 (22.9)	
Other	83 (2.6)	14 (1.7)	69 (2.9)	
Prefer not to answer	18 (0.6)	8 (1.0)	10 (0.4)	
**Number of comorbidities**				**<0.01**
Total number (0)	1420 (44.6)	399 (48.4)	1021 (43.2)	
Total number (1–2)	1286 (40.4)	331 (40.1)	955 (40.4)	
Total number (3–4)	402 (12.6)	79 (9.6)	323 (13.7)	
Total number (≥5)	79 (2.5)	16 (1.9)	63 (2.7)	
Cardiometabolic Comorbidities *				0.08
Yes	1239 (38.9)	300 (36.4)	939 (39.8)	
No	1948 (61.1)	525 (63.6)	1423 (60.3)	
**Vitamin D Status ^**				**<0.01**
Deficient	1481 (45.7)	424 (50.2)	1057 (44.2)	
Insufficient	947 (29.3)	237 (28.1)	710 (29.7)	
Optimal	759 (23.4)	164 (19.4)	595 (24.9)	
**Resided in a Rural Setting**				**<0.01**
Yes	946 (29.7)	156 (18.9)	790 (33.5)	
No	2227 (69.9)	665 (80.6)	1562 (66.1)	
**Region**				**<0.01**
West	812 (25.5)	356 (43.2)	456 (19.3)	
Midwest	436 (13.7)	171 (20.7)	265 (11.2)	
Southeast	1939 (60.8)	298 (36.1)	1641 (69.5)	

* Cardiometabolic comorbidities include self-reported history of stroke, heart attack, cardiac conditions, high blood pressure, or diabetes; other comorbidities include asthma, COPD, HIV/AIDS, chronic kidney disease, liver disease, cancer or leukemia/multiple myeloma, obesity; ^ Vitamin D status-deficient—<20 ng/mL, insufficient—≥20 and <30 ng/mL, and optimal—≥30 ng/mL; Values in bold indicate *p* value ≤ 0.05.

**Table 2:** Relationships between cohort characteristics and vitamin D status (*n* = 3187).

Variables	Total	Vitamin D Status ^			*p*-Value
		Deficient (*n* = 1481)	Insufficient (*n* = 947)	Optimal (*n* = 759)	
	N (%)	N (%)	N (%)	N (%)	
**Age**					**<0.01**
18–39	889 (27.5)	474 (32.0)	236 (24.9)	161 (21.2)	
40–59	1354 (41.8)	590 (39.8)	411 (43.4)	332 (43.7)	
60+	995 (30.7)	417 (28.2)	300 (31.7)	266 (35.1)	
**Sex**					0.50
Female	1915 (60.1)	892 (60.2)	562 (59.4)	461 (60.7)	
Male	1260 (39.5)	582 (39.3)	383 (40.4)	295 (38.9)	
Other	3 (0.1)	1 (0.1)	0 (0.0)	2 (0.3)	
**Race and Ethnicity**					**<0.01**
Hispanic or Latino	1558 (48.9)	800 (54.0)	470 (49.6)	288 (37.9)	
Black, non-Hispanic	802 (25.2)	329 (22.2)	239 (25.2)	234 (30.8)	
White, non-Hispanic	640 (20.1)	263 (17.8)	176 (18.6)	201 (26.5)	
Other	83 (2.6)	36 (2.4)	27 (2.9)	20 (2.6)	
Prefer not to answer	18 (0.6)	10 (55.6)	6 (33.3)	2 (11.1)	
**Number of Comorbidities**					**<0.01**
Total number (0)	1447 (44.7)	715 (48.3)	417 (44.0)	288 (37.9)	
Total number (1–2)	1304 (40.3)	573 (38.7)	388 (41.0)	325 (42.8)	
Total number (3–4)	408 (12.6)	158 (10.7)	127 (13.4)	117 (15.4)	
Total number (≥5)	79 (2.4)	35 (2.4)	15 (1.6)	29 (3.8)	
Cardiometabolic Comorbidities *					**<0.01**
Yes	1255 (38.8)	984 (66.4)	567 (59.9)	397 (52.3)	
No	1983 (61.2)	497 (33.6)	380 (40.1)	362 (47.7)	
**Resides in a Rural Setting**					**<0.01**
Yes	946 (29.7)	308 (20.8)	284 (30.0)	354 (46.6)	
No	2227 (69.9)	1165 (78.7)	658 (69.5)	404 (53.2)	
**Region**					**<0.01**
West	812 (25.5)	479 (34.3)	202 (22.9)	131 (14.4)	
Midwest	436 (13.7)	248 (17.8)	99 (11.2)	89 (9.8)	
Southeast	1939 (60.8)	668 (47.9)	582 (65.9)	689 (75.8)	

* Cardiometabolic comorbidities include self-reported history of stroke, heart attack, cardiac conditions, high blood pressure, or diabetes; other comorbidities include asthma, COPD, HIV/AIDS, chronic kidney disease, liver disease, cancer or leukemia/multiple myeloma, obesity; ^ Vitamin D status-deficient—<20 ng/mL, insufficient—≥20 and <30 ng/mL, and optimal—≥30 ng/mL; Values in bold indicate *p* value ≤ 0.05.

**Table 3:** Medical history associated with vitamin D status (deficient, insufficient, optimal) (*n* = 3187).

Variables	Total	Vitamin D Status ^			*p*-Value
		Deficient (*n* = 1481)	Insufficient (*n* = 947)	Optimal (*n* = 759)	
	N (%)	N (%)	N (%)	N (%)	
**Asthma**	327 (10.3)	150 (10.1)	88 (9.3)	89 (11.7)	0.25
**COPD**	102 (3.2)	45 (3.0)	25 (2.6)	32 (4.2)	0.16
**Diabetes**	597 (18.7)	247 (16.7)	171 (18.1)	179 (23.6)	**<0.01**
**History of Stroke**	74 (2.3)	26 (1.8)	23 (2.4)	25 (3.3)	0.07
**History of Heart Attack**	69 (2.2)	23 (1.6)	20 (2.1)	26 (3.4)	**0.02**
**High Blood Pressure**	978 (30.7)	383 (25.9)	309 (32.6)	286 (37.7)	**<0.01**
**Cardiac Condition**	179 (5.6)	65 (4.4)	49 (5.2)	65 (8.6)	**<0.01**
**HIV/AIDS**	10 (0.3)	5 (0.3)	2 (0.2)	3 (0.4)	0.78
**Chronic Kidney Disease**	40 (1.3)	17 (1.2)	14 (1.5)	9 (1.2)	0.76
**Liver Disease**	26 (0.8)	12 (0.8)	4 (0.4)	10 (1.3)	0.12
**Cancer or leukemia/multiple myeloma**	88 (2.8)	48 (3.2)	22 (2.3)	18 (2.4)	0.30
**Smoker**	295 (9.3)	141 (9.5)	76 (8.0)	78 (10.3)	0.25
**Obesity**	282 (8.9)	134 (9.1)	82 (8.7)	66 (8.7)	0.93

^ Vitamin D status-deficient—<20 ng/mL, insufficient—≥20 and <30 ng/mL, and optimal–≥30 ng/mL; Values in bold indicate *p* value ≤ 0.05.

**Table 4:** Serum total 25(OH)D stratified by SARS-CoV-2 infection status, demographic characteristics, and comorbidities (*n* = 3187).

	Total Vitamin D									
	Mean (SD)									
	Median									
	Total		Black Non-Hispanic		Hispanic/Latino		White Non-Hispanic		Other	
	*n*	ng/mL	*n*	ng/mL	*n*	ng/mL	*n*	ng/mL	*n*	ng/mL
All	3187	23.7 (13.9)	802	25.5 (14.9)	1558	21.9 (12.1)	640	26.3 (15.8)	83	23.7 (14.3)
		20.0		22.0		19.0		23.0		21.0
			**<0.01**							
**SARS-CoV-2 Infection**										
Positive	825	22.2 (13.2)	214	25.1 (15.6)	460	20.8 (11.2)	100	23.6 (15.6)	14	20.7 (11.1)
		19.0		21.0		18.0		19.0		17.5
Negative	2362	24.2 (14.1)	588	25.6 (14.6)	1098	22.3 (12.4)	540	26.8 (15.8)	69	24.4 (14.9)
		21.0		22.0		20.0		23.0		22.0
	**<0.01**		**<0.01**		**<0.01**		**<0.01**		**<0.01**	
**Age**										
18–39	871	21.8 (12.7)	131	23.7 (14.5)	516	20.4 (10.8)	169	23.8 (14.3)	24	28.4 (21.2)
		18.0		20.0		18.0		19.0		
40–59	1333	24.2 (14.5)	330	26.0 (15.4)	686	22.6 (13.1)	233	27.6 (17.4)	35	23.9 (10.2)
		21.0		22.0		20.0		24.0		24.0
60+	983	24.7 (14.0)	341	25.6 (14.5)	356	22.6 (11.6)	238	26.8 (15.1)	24	19.0 (9.1)
		21.0		22.0		20.0		24.0		16.5
	**<0.01**		**<0.01**		**<0.01**		**<0.01**		**<0.01**	
**Sex**										
Female	1915	23.8 (13.9)	499	25.2 (15.2)	961	22.2 (12.0)	359	26.0 (15.6)	48	22.9 (11.1)
		20.0		21.0		19.0		22.0		22.5
Male	1260	23.7 (14.0)	303	25.8 (14.3)	594	21.5 (12.3)	278	26.7 (16.2)	35	24.9 (18.0)
		20.0		23.0		19.0		23.0		20.0
	**<0.01**		**<0.01**		**<0.01**		**<0.01**		**<0.01**	
**Region**										
West	812	19.3 (10.7)	164	19.2 (8.9)	477	19.2 (10.8)	116	19.0 (10.0)	31	23.7 (18.5)
		17.0		17.0				16.0		20.0
Midwest	436	20.7 (13.3)	62	21.2 (11.4)	222	19.1 (11.6)	80	24.4 (16.6)	10	25.4 (14.4)
		17.0		18.0		16.0		20.5		23.0
Southeast	1939	26.2 (14.6)	571	27.8 (15.9)	859	24.1 (12.5)	444	28.6 (16.3)	42	23.4 (10.7)
		23.0		25.0		21.0		25.0		21.0
	**<0.01**		**<0.01**		**<0.01**		**<0.01**		**<0.01**	
**Comorbidities**										
Total number (0)	1420	22.4 (12.9)	227	22.9 (12.2)	844	21.7 (12.3)	243	24.5 (14.9)	45	24.0 (17.5)
		19.0		20.0		19.0		20.0		20.0
Total number (1–2)	1286	24.3 (14.2)	375	25.8 (15.6)	580	22.3 (12.2)	264	26.9 (16.2)	30	24.2 (9.0)
		21.0		22.0		20.0		23.5		24.5
Total number (3–4)	402	25.6 (14.5)	166	27.1 (13.7)	118	20.7 (9.8)	105	28.7 (16.4)	8	20.6 (11.4)
		23.0		25.0		18.5		24.0		19.0
Total number (≥5)	79	28.3 (19.7)	34	30.6 (24.0)	16	25.3 (13.2)	28	26.9 (17.6)	-	-
		23.0		23.5		23.5		19.5		
	**<0.01**		**<0.01**		**<0.01**		**<0.01**		**<0.01**	
Cardiometabolic Comorbidities *										
Yes	1239	25.7 (15.0)	467	27.3 (15.6)	459	22.5 (12.0)	262	28.7 (17.1)	28	24.4 (10.0)
		22.0		24.0		20.0		25.0		24.5
No	1948	22.4 (13.1)	335	22.9 (13.4)	1099	21.6 (12.1)	378	24.7 (14.7)	55	23.4 (16.2)
		19.0		20.0		19.0		21.0		20.0
	**<0.01**		**<0.01**		**<0.01**		**<0.01**		**<0.01**	

* Cardiometabolic comorbidities include self-reported history of stroke, heart attack, cardiac conditions, high blood pressure, or diabetes; other comorbidities include asthma, COPD, HIV/AIDS, chronic kidney disease, liver disease, cancer or leukemia/multiple myeloma, obesity; Vitamin D levels among participants with missing data on study characteristics were not included in comparisons of categories. Vitamin D levels among participants with missing data were compared to the distribution of vitamin D in the entire study population; Values in bold indicate *p*-value ≤ 0.05.

**Table 5:** Associations between demographic factors, vitamin D Levels and the combined molecular and serologic assessment of SARS-CoV-2 status (*n* = 2699).

Variables		Adjusted Odds Ratio	95% Confidence Interval
**Age**		**0.99**	**0.98–0.99**
**Race/Ethnicity**			
	Hispanic or Latino	**2.01**	**1.56–2.59**
	Black, non-Hispanic	**1.91**	**1.44–2.53**
	White, non-Hispanic	Reference	Reference
**Vitamin D Level**	-	**0.88**	**0.79–0.98**

Values in bold indicate *p* value ≤ 0.05.

**Table 6:** Associations between location, medical history, demographic factors, vitamin D levels and the combined molecular and serologic assessment of SARS-CoV-2 status (*n* = 2699).

Variables		Adjusted Odds Ratio	95% Confidence Interval
**Region ^**			
	Midwest	**4.04**	**3.08–5.28**
	West	**4.51**	**3.65–5.57**
	Southeast	Reference	Reference
**Resides in a rural setting ^**			
	Yes	**0.45**	**0.35–0.58**
	No	Reference	Reference
**Cardiometabolic Outcomes ^**			
	Yes	1.03	0.84–1.26
	No	Reference	Reference
**Number of Comorbidities**			
	Total number (1–2)	1.04	0.85–1.26
	Total number (3–4)	0.74	0.54–1.03
	Total number (≥5)	0.86	0.45–1.63
	Total number (0)	Reference	Reference

Each row indicates a separate model that was adjusted for age, race/ethnicity, and vitamin D levels; values in bold indicate *p*-value ≤ 0.05; ^ vitamin D did not remain statistically significant in the minimally adjusted model.

## Data Availability

MRCIS cohort data is available upon request. Requests should be sent to salesci@nmqf.org.

## Supplementary Materials

The following supporting information can be downloaded at: https://www.mdpi.com/article/10.3390/v16040639/s1, Figure S1. MRCIS Cohort Participant Flow; Table S1. Associations between demographic factors, vitamin D Status and the combined molecular and serologic assessment of SARS-CoV-2 status (n = 2699).
