# Supplementary material for: Vitamin D Levels and SARS-CoV-2 Infection among Medically Underserved Populations in the Minority and Rural Coronavirus Insights Study
Source: Viruses. 2024 Apr 19;16(4):639. doi: 10.3390/v16040639 (PMC11053904; doi:10.3390/v16040639)
Supplement: Supplementary file 1 [file viruses-16-00639-s001.zip › viruses-2931807-supplementary.pdf]

viruses

MDPI

Supplementary Materials

Vitamin D Levels and SARS-CoV-2 Infection among Medically Underserved Populations in the Minority and Rural Coronavirus Insights Study

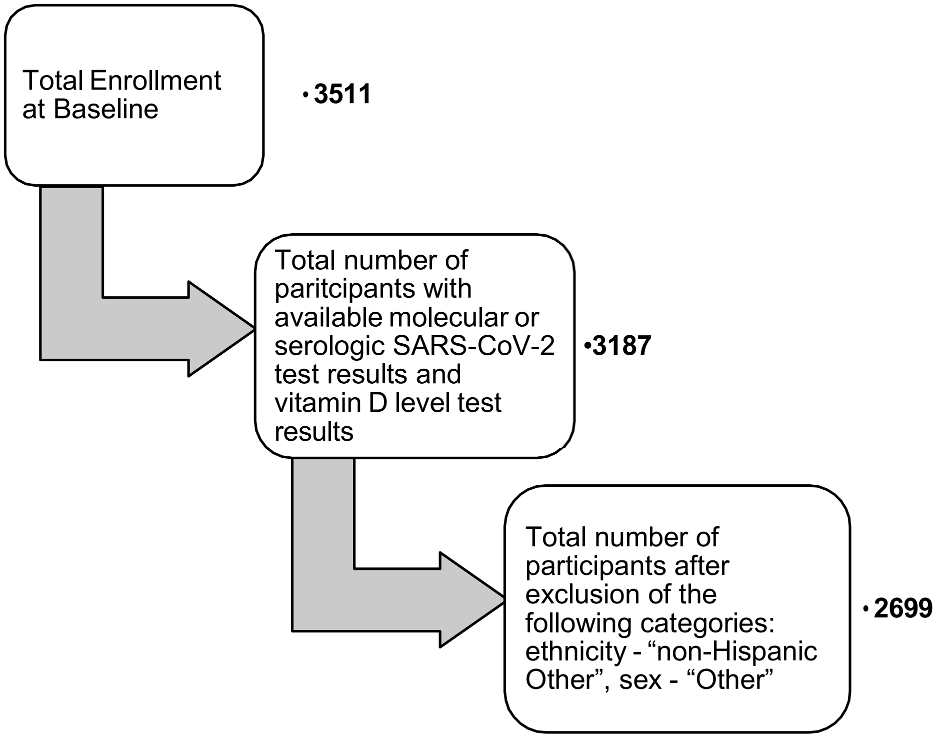

Figure S1. MRCIS Cohort Participant Flow.

Table S1. Associations between demographic factors, vitamin D Status and the combined molecular and serologic assessment of SARS-CoV-2 status (*n* = 2699).

Variables		Adjusted Odds Ratio	95 % Confidence Interval
**Age**		***0.99***	***0.98–0.99***
**Race/Ethnicity**			
	Hispanic or Latino	***2.03***	***1.57–2.62***
	Black, non-Hispanic	***1.92***	***1.44–2.54***
	White, non-Hispanic	*Reference*	*Reference*
**Vitamin D Status ^**	Optimal	0.82	0.64–1.07
	Insufficient	0.86	0.71–1.04
	Deficient	*Reference*	*Reference*

^ Vitamin D status—deficient—<20 ng/mL, insufficient—≥20 and <30 ng/mL, and optimal—≥30 ng/mL.
